# The Guanine-Quadruplex Structure in the Human *c-myc* Gene's Promoter Is Converted into B-DNA Form by the Human Poly(ADP-Ribose)Polymerase-1

**DOI:** 10.1371/journal.pone.0042690

**Published:** 2012-08-06

**Authors:** Anna Fekete, Erzsebet Kenesi, Eva Hunyadi-Gulyas, Hajnalka Durgo, Barbara Berko, Zsuzsanna A. Dunai, Pal I. Bauer

**Affiliations:** 1 Department of Medical Biochemistry, Semmelweis University, Budapest, Hungary; 2 Laboratory of Proteomics, Biological Research Center, Hungarian Academy of Science, Szeged, Hungary; 3 Department of Pathogenetics, National Institute of Oncology, Budapest, Hungary; Institut de Génétique et Développement de Rennes, France

## Abstract

The important regulatory role of the guanine-quadruplex (GQ) structure, present in the nuclease hypersensitive element (NHE) III_1_ region of the human *c-myc* (h *c-myc*) gene's promoter, in the regulation of the transcription of that gene has been documented. Here we present evidences, that the human nuclear poly(ADP-ribose)polymerase-1 (h PARP-1) protein participates in the regulation of the h *c-myc* gene expression through its interaction with this GQ structure, characterized by binding assays, fluorescence energy transfer (FRET) experiments and by affinity pull-down experiments *in vitro*, and by chromatin immunoprecipitation (ChIP)-qPCR analysis and h c-myc-promoter-luciferase reporter determinations *in vivo*. We surmise that h PARP-1 binds to the GQ structure and participates in the conversion of that structure into the transcriptionally more active B-DNA form. The first Zn-finger structure present in h PARP-1 participates in this interaction. PARP-1 might be a new member of the group of proteins participating in the regulation of transcription through their interactions with GQ structures present in the promoters of different genes.

## Introduction

The GQ non-B-DNA structure is built up from two or more parallel guanine-tetrad layers, each containing four guanine units held together by Hoogsteen base pairing [1]. The GQ structure first has been found in telomers but recently *in silico* estimations predict their total number in the whole genome to be around 350 000 units [2]. Their distribution in the genome is not random, higher proportions are found in the promoter or untranslated region (UTR) of genes compared to exons of genes or intergenic regions. Genes with high biological importance as *c-myc*, *K-* and *N-ras*, *ret*, *met*, *myb*, *ets1*, *vegf* or *hif1A* contain GQ structures in their promoters [3]–[7]. Experiments have proven their existence and structure and their modus operandi in the regulation of transcription *in vitro*, but much less is known about their role and importance in the regulation of transcription *in vivo*. The most extensively studied GQ containing promoter is that of the h *c-myc* gene [3]. The c-MYC protein is one of the most important transcriptional factors, participating in the regulation of 10% of all genes and regulating such important biochemical processes as cell growth, differentiation and cell death. Its mutated form or its upregulation is found in most cancers [8]. The regulation of the *myc* gene activity is very complex, happens at multiple levels in transcription and translation too. In the promoter region of the *c-myc* gene there are multiple nuclease hypersensitive sites. The NHE III_1_ site contains guanine rich segments, which have the ability to form isomorphic GQ structures, which are in equilibrium with the double-stranded B-DNA form of that region [3], [9]. The protruding GQ structure and the I-motif formed on the opposite strand keep the two DNA strands separated and prevent the formation of the basal transcriptional complex. When this promoter region is in B-DNA form the transcription can be initiated [6]. The regulation of GQ formation as well as the protein complex which helps its formation or smoothes the GQ form out is continuously getting explored. Certain proteins (nucleolin, NMD23-H2, hnRNP K, CNBP, MAZ) [10]–[14] are known to bind GQ structures, influencing either the formation or the removal of this knob-like structure. Hurley *et al.* have shown that 90% of the regulation of the *c-myc* gene happens at the GQ level [3], [5]–[7].

The PARP-1 protein is a pleiotropic modifier of biological processes, participating in the regulation of transcription, replication and DNA-repair, serves as regulator of cell death, and also is involved in keeping up the integrity of the genome etc. [15]–[18]. Recently, it has been shown to influence transcription together with topoisomerases [19]. These multilevel activities of PARP-1 are manifested through its protein-DNA, protein-protein interactions and also by its ability to synthesize and attach poly(ADP-ribose) (PAR) moieties to target proteins including itself [15], [16], [19]–[22]. Its enzyme activity is regulated by its binding to DNA, recognizing single- and double-stranded breaks and also by interacting with non-B-DNA structures. Loops, 3′ and 5′ prime overhangs, hairpin or cruciform structures were shown to associate with PARP-1, increasing its enzymatic activity [23], [24]. Recently PARP-1 binding to the GQ structures present in *K-ras* and *c*-*kit* promoters were shown [11], [24].

In this study we show that PARP-1 interacts with the GQ structure forming DNA element of the NHE III_1_ promoter segment of the human *myc* gene both *in vitro* and *in vivo* and through this interaction is influencing the transcriptional activity of the *c-myc* gene.

## Results

In vitro, h PARP-1 binds to and is activated by the GQ structure present in the promoter region of the h c-myc gene. PARP-1 present in HeLa cell extracts, shows binding to both single-stranded and double-stranded forms of wild type h myc GQ DNA.

**Figure 1:**
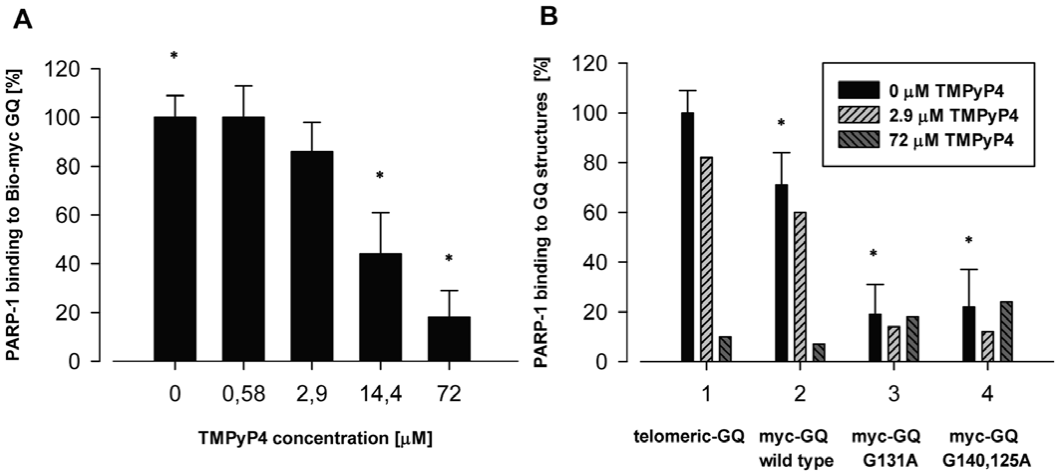
*In vitro* binding of h PARP-1 to the h *c-myc* GQ structure and the effect of TMPyP4 on that binding. Figure 1A Effect of TMPyP4 on the binding of PARP-1 to the *c-myc* GQ structure. 0.45 pmol aliquots of h PARP-1 were incubated with 5 pmols of 5′-biotine end-labeled wild type h *c-myc* GQ oligonucleotide (wild type GQ, 5′-biotin-TGG GGA GGG TGG GGA GGG TGG GGA AGG) in the absence and in the presence of different concentrations of the cationic porphyrin compound TMPyP4 as described in the Materials and Methods. Binding is expressed as % of binding measured in the absence of competing TMPyP4 and is shown on the ordinate. TMPyP4 concentrations (0, 0.58, 2.9, 14.4, and 72 µM) are shown on the abscissa. Asterisks represent samples where the extent of binding are significantly different (p<0.05, Student t-test). Figure 1B *In vitro* binding of h PARP-1 to various GQ structures and the competing effect of TMPyP4 on that binding. 0.45 pmol aliquots of h PARP-1 were incubated either with 5 pmols h telomeric GQ structure (h-telomeric GQ, 5′-biotin-TTA GGG TTA GGG TTA GGG TTA GGG) or with wild type *c-myc* GQ structure (wild type GQ, 5′-biotin-TGG GGA GGG TGG GGA GGG TGG GGA AGG) or its mutants (mutant-1 GQ, 5′-biotin-TGG GGA GGG TG**A** GGA GGG TGG GGA AGG, mutant-2 GQ, 5′-biotin-TG**A** GGA GGG TGG GGA G**A**G TGG GGA AGG). The sites of mutations are shown in bold. Binding assay was carried out as described in Materials and Methods. Ordinate shows the binding of PARP-1 to GQ structures (telomeric GQ, *c-myc* GQ, G-131A and G-140, -126A) expressed in percentage. The amount of PARP-1 bound to telomeric GQ is taken as 100%. Insert shows the applied concentrations of TMPyP4. Asterisks represent samples where the difference in binding is significantly different (p<0.05).

PARP-1 is known to recognize the murine K-ras and the human c-kit GQ structures [11], [24]. To characterize the possible interaction between the h PARP-1 protein and the h c-myc GQ structure a binding assay was developed. As it is shown in Fig. 1A, PARP-1 binds to the wild type h c- myc GQ structure, while it displays only a very limited binding activity towards to the mutated h myc GQ sequences, the standard deviation of the binding assay is roughly equal to the measured values of binding (Fig. 1B). The wild type h c-myc GQ oligonucleotide has a sequence identical with the DNA sequence of the h c-myc gene's promoter region, spanning between positions −142 to −116. In the mutant-1 h c-myc GQ sequence a guanine base is replaced with an adenine base at position -131. The mutant-2 h c-myc GQ is a double mutant of the wild type of sequence bearing two guanine to adenine replacements, at positions −140 and at −126. The human telomeric GQ-DNA also displayed PARP-1 binding. The cationic porphyrine TMPyP4 binds to the highest and the lowest guanine tetrad layers and stabilizes the GQ structure. Because PARP-1 was shown to bind to the base part of the cruciform structure [25], we carried experiments to show, whether h PARP-1 binding to the wild type h c-myc GQ structure is influenced by TMPyP4. As it is shown in Fig. 1A, TMPyP4 competes out the binding of PARP-1 to the GQ structure.

**Figure 2:**
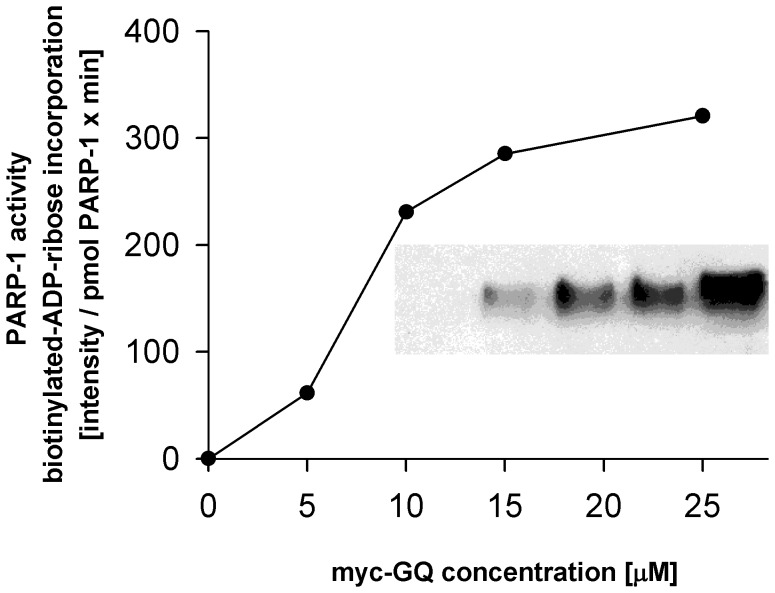
*In vitro* effect of wild type h *c-myc* GQ structure on the enzymatic activity of h PARP-1. PARP-1 (1 pmol) was incubated with 6-biotin-17-NAD (75 µM) in the presence of various concentrations of *c-myc* GQ oligonucleotides for 10 minutes at 23°C. At the end of incubation an equal volume of Laemmli sample buffer was admixed, boiled samples were electrophoresed (10% SDS-PAGE), and transblotted onto nitrocellulose sheet and the aspecific binding sites were blocked by incubating the blot in a solution containing 3% fat free milk dissolved in PBS. This was followed by an incubation of the blot with HPO conjugated streptavidine (1 µg/ml) for an hour. Bound biotin-ADP-ribose was detected by ECL. The fluorogram shown in the insert (*c-myc* GQ concentrations used in the assays are from left to right: 0, 5, 10, 15 and 25 µM). Spot intensities were quantitated using a Bio-Rad gel-analyzer system. Ordinate shows the incorporation of biotin-ADP-ribose in pixel intensity/pmol PARP-1× min units while the applied GQ concentrations are shown on the abscissa. One representative experiment is shown from three independent experiments.

The 50% inhibitory concentration of TMPyP4 was around 8–12 µM (Fig. 1A). The PARP-1 bound *c-myc* GQ increased the enzymatic activity of the protein in a concentration-dependent way and GQ was a better activator of the enzyme than single-stranded DNA or RNA (Fig. 2 and Table S1) [26]. The *c*-*myc* GQ concentration resulting in 50% activation of the enzymatic activity PARP-1 enzymatic activity was around 10 µM (Fig. 2).

To show that h PARP-1 binding to h *c-myc* GQ structure also exists in cell extracts too, affinity pull-down experiments using either biotinylated-h-c-*myc* GQ single-stranded oligonucleotide, or its B-DNA-form, or biotinylated dA/dT double-stranded DNA as baits, were carried out. Cell extracts were isolated either from logarithmically growing or 1% FBS starved HeLa cells. Pull-down experiments were carried out as described in the Materials and Methods section. Fig. S1A shows the protein profiles of GQ binding proteins and Table S2 lists the sequenced proteins, which could be sorted into three groups. Group I proteins are known to bind to GQ structures, PARP-1 [11], nucleolin [13], [27] and heterogeneous nuclear ribonucleoprotein (hnRNP) isoforms [14], [28] belong to this family. PARP-1 was found in two of the sequenced samples, independently whether extracts were made from growing or starving cells, when proteins were identified using LC-MS/MS.. The PARP-1 protein was bound both to the single-stranded and to the double-stranded forms of h *c-myc* GQ structure containing baits as pictured by immunoblotting too (Fig. S1B). It is interesting to note that the same fractions (lane 4 and 5 in Fig. S1A) tested positive for the presence of Topoisomerase I too. Group II proteins are known to recognize short double-stranded RNA and DNA molecules and are participating in their unfolding. ATP-dependent DNA helicase II, growth regulated nuclear p68 protein, ras-GTP-ase activating protein binding protein which recognizes SH3 domain and DEAH-box polypeptide 36 isoform are in this category [29]–[32]. To the third group those proteins are enlisted which are hard to connect to the transcription of *c-myc* gene. Spectrin, non-erythroid alpha spectrin belongs to this group.

In vivo, h PARP-1 binds to the promoter region of the h c-myc gene shown by ChIP-qPCR experiments and PARP-1 activates the transcription of the c-myc gene.

In our further experiments we have chosen experimental conditions where the *c-myc* gene either is expressed or is silenced, and therefore we considered the *c-myc* promoter being either in B-DNA or in GQ form. It is claimed that the GQ structure provided switch mechanism plays an 80–90% role in the regulation of transcription of the *c-myc* gene [4], [7], [33], [34]. In HL60 (human promyelocytic leukemia cell line) cells *myc* gene is highly expressed from multiple gene copies, but when induced to differentiate the *myc* gene is rapidly down-regulated, the *c-myc* mRNA level is rapidly decreasing. After 24 hours the copy number of *myc* gene starts to decrease too [35]. On the other hand, in serum starved HeLa cells the *myc* gene is down-regulated and in case of serum readdition the level of expression is radically increasing. First we have proven that under the selected conditions the expression of the *myc* gene was changing as expected by using RT-qPCR experiments. In HeLa cells the expression of *c-myc* and *cyclin D* genes being very sensitive markers of cell cycle promotion were upregulated after an hour of serum addition, while the expression of *parp-1* and *gapdh* genes did not change too much and were behaving as expected for housekeeping genes. (Fig. S2). Benzamide, an inhibitor of PARP-1 did not influence the expression of *c-myc* and *parp-1* genes in logarithmically growing HeLa cells (data not shown) indicating that the enzyme activity of PARP-1 does not influence the transcription of those genes. In HL60 cells benzamide induces differentiation and as a consequence of differentiation into granulocytes the *myc* expression and *PARP-1* expression are both decreasing (not shown). Granulocytes are one of the rare cell types which does not have PARP-1 protein [36]. Starting with equal numbers of treated and non-treated cells, ChIP-qPCR experiments were carried out. A typical qPCR curve set of the ChIP-qPCR assay is shown in Fig. S3, obtained from growing and from differentiation induced HL60 cells. Contrary to our expectations it was always found that we got more h *c-myc* promoter DNA immunoprecipitated in experimental setups, where the GQ structure was expected to be in the B-DNA form (Table 1 and Fig. S3). The PCR products were homologous with a melting point of 81°C. When the PCR products were DNA sequenced their nucleotide sequences were identical. Experimental variations of ChIP experiments were also conducted, where, before formaldehyde crosslinking, cells were treated with the cell-permeable protein-protein crosslinker ethylene glycol bis(succinimidyl succinate) (EGS) [37]. These experiments resulted in similar observations; more PARP-1 was bound to the B-DNA structure compared to the GQ structure. When antibodies developed against other PARP-1 interacting proteins were applied in the immunoprecipitation process, we found that the Topoisomerase I antibody was also able to pull down the NHE III_1_ region of the *c-myc* promoter (data not shown).

**Table 1:** ChIP-qPCR analysis of *in vivo* binding of h PARP-1 to the NHE III_1_ region present in the promoter of the h *c-myc* gene, and which is presumably either in the GQ form or in the double stranded B-DNA form.

Cell line used	Treatment	Antibody	c1/c2 = 2^n2-n1^
HL60	1.2% DMSO, 2 hour	PARP-1	6.6±0.8
HL60	1.2% DMSO, 24 hours	PARP-1	9.75±1.2
HeLa	10% FBS addition, 1 hour	PARP-1	22±8

ChIP-qPCR analysis was carried out as described in the Materials and Methods section using a polyclonal antibody raised against PARP-1 (Santa Cruz, H250; 2 µg/extract of one million cells) as precipitation agent. The treatment modalities and the ratio of PARP-1 bound promoter DNAs, isolated from differently treated cells (HeLa and HL60), and amplified by qPCR and calculated from the fluorescence of the Eva-Green complexes formed with the double-stranded PCR products, are shown. The c1/c2 values represent the ratio of the concentrations of PARP-1 bound *c-myc* promoter DNAs present in the ChIP products obtained from the treated (1) and from the non-treated (2) cell populations and calculated using the c1/c2 = 2^n2 – n1^ formula, where n1 and n2 are the number of PCR cycles needed to reach the same fluorescence value in the logarithmic phase of the PCR curves.

**Figure 3:**
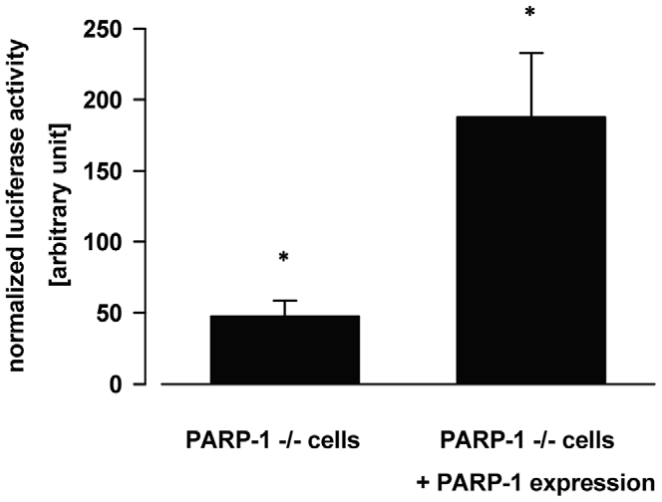
The expression of h PARP-1 *in vivo* increases the luciferase enzyme activities assayed in Del4 reporter plasmid transfected PARP −/− MEF cells. Logarithmically growing PARP −/− MEF cells were transfected with Del-4 plasmid together with the pcDNA3.1-*beta-galactosidase* expressing plasmid and in the absence or in the presence of h PARP-1 expression (pcDNA3.1-*parp-1* plasmid). After two days of incubation cells were splitted into six-well plates and grown for a day further. Than cells were harvested, lysed and their luciferase and beta-galactosidase enzyme activities were determined. The luciferase enzyme activities were normalized for beta-galactosidase activities and are shown in the figure. Asterisks show significant difference in the reporter enzyme activities between the two pools of sample (p<0.05).

Because ChIP experiments suggested that PARP-1 binds to both forms of the h *c-myc* promoter, we experimentally tested how PARP-1 is influencing the promoter activity of the h *c-myc* gene. Therefore reporter assays were set up as described in the Materials and Methods section. Three days after transfections, cell lysates were prepared and their luciferase and beta-galactosidase activities were assayed. Fig. 3 shows that as a consequence of h PARP-1 expression the beta-galactosidase normalized luciferase activities were increased from 47.4±11.1 units to 187.5±45.2 units representing a strong and significant increase in the h *c-myc* promoter activity of the reporter plasmid.

H PARP-1 accelerates the in vitro conversion of the single-stranded form of h c-myc GQ into its B-DNA form in the presence of the complementary DNA strand. Zn-finger-I of PARP-1 participates in this action.

Our results indicated that PARP-1 is a positive modulator of h *c-myc* gene expression by binding both *in vitro* and *in vivo* to the GQ structure present in the h *c-myc* gene promoter. We designed FRET experiments to show whether h PARP-1 participates in the switching mechanism of h *c-myc* gene expression. Therefore a 27-mer oligonucleotide was synthesized covering the base sequence of the promoter of the h *c-myc* gene, from bases −142 to −116 which sequence covers the GQ forming part of the promoter and has a 5′ fluoresceine and 3′ tetramethylrhodamine labeling (see Materials and Methods). This oligonucleotide displays FRET when curled up into the GQ form and is loosing FRET when either is annealed to the complementary strand or is converted into a coiled coil single-stranded form. We measured the fluorescence spectrum of this deoxy-oligonucleotide in the presence of 100 mM KCl where the GQ form is favored. It is shown in Fig. S4, that a significant FRET peak was observed at 585 nm. At a fixed F-*myc* GQ-R concentration and in the presence of a tenfold molar excess of the complementary strand, due to double stranded DNA formation, the FRET peak disappeared in a time frame of 15 minutes. Applying this concentration ratio of the two deoxy-oligonucleotides, a semi-kinetic model has been created providing an experimental tool to study the effect of added proteins on the annealing process. The FRET intensity of F-*myc* GQ-R was measured at 0 minute, then the protein was admixed and FRET spectra were recorded at different time points. The rate of the decrease of the FRET peak height during one minute was shown as a percentage of the original peak height (Table 2). We have shown that h PARP-1 catalyzes the annealing reaction in a concentration-dependent way. When truncated mutants of h PARP-1 were tested, the importance of the first zinc-finger structure was observed. In the absence of the first Zn-finger the rate of annealing reaction was not increased over the control value. Contrary to that, the absence of the second Zn-finger did not influence the rate increasing effect of PARP-1. PARylated h PARP-1 did not accelerate the annealing process. It is also interesting to mention that human Topoisomerase I, either alone or in combination with h PARP-1 could increase the rate of annealing in a non-synergistic way, but we have to consider the option too, that the FRET peak was decreasing due to endonucleotidic splitting of the GQ structure by h Topoisomerase I. Other PARP-1 interacting proteins did not influence the rate of annealing (Table 2).

**Table 2:** Influence of nuclear proteins on the kinetics of unfolding of the h *c-myc* GQ structure, assayed by measuring the decrease in FRET activities in the presence of the complementary DNA strand.

Experiment no.	Protein added	Δ FRET activity (%)*	n‡
1.	Complementary strand alone	14±4	4
2.	1. and 0.1 µg PARP-1	20±4	2
3.	1. and 0.5 µg PARP-1	35±5	4
4.	1. and 0.5 µg PARP-1 ΔZn-I	11±1	3
5.	1. and 0.5 µg PARP-1 ΔZn-II	28±6	3
6.	1. and 0.5 µg PARP-1 ΔZn-I&II	13±3	3
7.	1. and 0.5 µg PARylated PARP-1	16±3	2
8.	1. and 0.05 µg TOPO I	27±2	2
9.	1. and 0.05 µg TOPO I and 0.5 µg PARP-1	45±7	2
10.	1. and 1 µg spermin	12±6	2
11.	1. and 1 µg histone H1	15±7	2
12.	1. and 1 µg P53	14±8	2

* decrease in FRET activity during one minute (% of decrease in the maximal height of the FRET peak measured at 0 time).

‡ number of experiments.

F-*myc* GQ-R double labeled FRET oligonucleotide (25 pmol) was incubated either alone or with the tested proteins for one minute in a volume of 50 µl, than its FRET spectra was taken (0 min value). The complementary, antiparallel oligonucleotide to the GQ structure (in a tenfold molar excess) was admixed and FRET spectra was recorded at 1, 3 and 5 minutes after annealing has been initiated. The kinetics of the decrease of the FRET peak heights were calculated and shown as ΔFRET peak value during one minute of annealing as percentage of the zero minute FRET peak height values. Excitation was at 485 nm and emissions were recorded between 500–650 nms.

**Figure 4:**
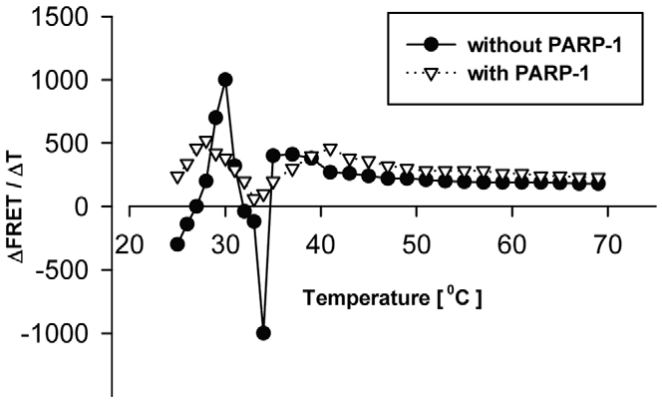
PARP-1 modifies the effect of increasing temperature on the FRET activities of the F-*c-myc* GQ-R molecule. FRET melting curves were taken in the absence or in the presence of 1 µg h PARP-1 and using a 2°C/2 min temperature gradient. Excitation was at 485 nm and emission was recorded at 585 nm. From the recorded data ΔFRET/ΔT values were calculated and plotted (ordinate) versus the applied temperatures (abscissa).

The *c-myc* GQ structure is one of the several isomorphic structures which are not characterized in details [38]–[41]. When those are transformed into each other, their oligonucleotide chains make movements, during which their two ends might get closer or move further from each other. The FRET method can follow these movements being the FRET intensity a function of the distance of the two fluorophores. Therefore we followed the FRET intensity changes during the melting of the single-stranded h *c-myc* GQ DNA structure in the absence and in the presence of h PARP-1. The temperature of the sample holding cuvette was increased with a constant rate (2°C/2 min) and the FRET values were recorded. From the data a secondary differential plot has been created and the ΔFRET/ΔT values were spotted versus the temperature. At Fig. 4 large oscillatory changes in the ΔFRET/ΔT values were seen in the first part of the curve between 25 and 35°C and which presumably were the consequence of conversions of certain isomorphic structures into another, different isomorphic form. The second part of the curve was nearly parallel with the abscissa, meaning a continuous decrease of the FRET as temperature was gradually increasing. In the presence of h PARP-1 the amplitude values of changes were decreased and the curve was smoothed out. PARP-1 might have served as a sink and stabilized one of the isomorphs. In the presence of the complementary chain this isomorphic structure might be converted directly either into the B-DNA form or with the help of other transcriptional factors into the preinitiation complex.

## Discussion

In this publication we present experimental evidence suggesting that h PARP-1 participates in the regulation of the transcription of the h *c-myc* gene.

We developed an *in vitro* binding assay which shows that h PARP-1 binds to the wild type GQ structure of the h *c-myc* promoter and has only a limited binding capacity towards the mutated ones having G to A exchanges (Fig. 1A and Fig. 1B) The cationic porphyrin TMPyP4 is known to bind to the stacked guanine-tetrad layers of the wild type h *c-myc* GQ structure by π-π interactions and by ionic interactions shown by NMR spectroscopy and by binding assays [42], [43]. TMPyP4 treatment of different cell lines reduces their *myc* expression through the stabilization of the GQ structure, when applied in a concentration between 20–100 µM [3], [5], [44]–[46]. TMPyP4 in a concentration dependent manner decreased the binding of h PARP-1 to the wild type of h *c-myc* GQ structure (Fig. 1A). The binding of TMPyP4 to the wild type h *c-myc* GQ structure is disrupted by G to A exchange mutations which distorted the flat structure of guanine-tetrad layers [3], [4]. The GQ structure is organized from the five guanine stretches present in the NHE III_1_ region by selecting four different ones to create various isomorphic GQ structures [9]. Experiments indicate that the basket form is the thermodynamically favored form, while the kinetically favored form is the chair one [46]. According to authors the chair form is the biologically relevant form [38]. The binding of TMPyP4 molecules to these structures converts the mixed parallel forms partly into antiparallel GQ isomorphic structures [41]. The IC_50_ value of our binding assay is relevant to the concentration of the drug used to reduce *myc* expression *in vivo* in various cell lines especially if we consider that TMPyP4 also shows non-specific DNA binding too [47]. Binding to the GQ structure increases the enzymatic activity of PARP-1 although to a smaller extent than the activation induced by breaks on DNA (Fig. 2, Table S1). In our experiments poly(ADP-ribos)ylated PARP-1 did not accelerate the rate of annealing of the two DNA strands (Table 2). We carried out pull-down experiments using cell extracts made from growing or serum starving cells and using single-stranded h *c-myc* GQ structure or its B-DNA form as baits. Aliquots of the eluates were SDS-PAGE electrophoresed and colloid Coomassie Blue-stained. Based on the protein profile obtained (Fig. S1A) selected bands were cut out and mass-spectra sequenced (Table S1). PARP-1 was found both in the eluates of the GQ form and that of the B-DNA form. This has been shown by immunoblotting too. In both fractions the presence of Topisomerase I was also shown (Fig. S1B). Between the sequenced proteins we found proteins already known to interact with GQ structures (Table S2). Nucleolin is a nucleolar phosphoprotein [4], [13], [27] participating among others in regulation of cell proliferation, transcription and also in the process of apoptosis. It is known to induce the formation of *c-myc* GQ structure from single-stranded DNA and also is known that nucleolin stabilizes the already formed GQ structure. The binding of nucleolin to different GQ structures has also been documented [27]. Different isoforms of hnRNP (R, M, and A1) have been found in the extracts of HeLa cells grown in 10% FBS containing medium and were hooked to the *c-myc* GQ structure. While these proteins were not known up till now to interact with *c-myc* GQ structure, another member of this family, hnRNP K is known to interact with the *c-myc* gene promoter participating in serum-induced activation of *c-myc* transcription [14]. HnRNP K specifically binds to single-stranded nucleic acids, in the case of *c-myc* promoter, to the pyrimidine rich strand of the promoter [28]. The interaction of other sequenced proteins with the h *c-myc* promoter has not been described yet. The DEAH-box polypetide isoform is known to recognize guanine-tetrads and it is responsible for the degradation of the guanine-tetrad structure [30]. The participation of hnRNP A1 in the regulation of the *K-ras* promoter has been documented as a protein which helps the unwinding of the GQ structure. Heat shock proteins (HSP) can participate in the regulation of transcription, but their (HSP70 and HSP90) direct interaction with GQ has not been described (Table S2.).

To characterize the interaction of PARP-1 with the promoter of the h *c-myc* gene *in vivo*, ChIP-qPCR experiments were carried out in cells kept under conditions where the *myc* gene is either active or it is turned off. Using RT-qPCR experiments we have first shown that the expression of the selected genes, *myc*, *cyclin D*, *parp-1* and *gapdh* followed the expected trends both in HeLa (Fig. S2) and in HL60 cells (not shown). In the ChIP-qPCR experiments using two different PARP-1 antibodies, to our surprise preferential binding of PARP-1 to the B-DNA form of the *c-myc* promoter was observed (Fig. S3 and Table 1). The same effect was observed when the protein crosslinking agent EGS was used before formaldehyde treatment. In order to further analyze the effect of h PARP-1 on the expression of the h *c-myc* gene *in vivo*, PARP-1 was expressed in rapidly growing PARP-1 −/− MEF cells transfected with the Del-4-luciferase reporter plasmid. This type of assay is frequently used to characterize the *in vivo* role of various GQ structures in the regulation of different genes e.g. *myc*, *kit*, *ras* [3], [5], [11], [46]. As a consequence of PARP-1 expression a very significant fourfold increase of the luciferase activity was observed (Fig. 3). From these results and that of the ChIP-qPCR experiments we surmised that PARP-1 binds to the GQ structure and participates in its conversion to the transcriptionally active B-DNA form.

A semi-kinetic FRET based experimental approach was designed, where annealing of the h *c-myc* GQ structure with its complementary oligonucleotide into their B-DNA form was followed by observing the decrease of FRET activity (Fig. S4). As Table 2 shows, h PARP-1 increases the rate of annealing in a concentration-dependent manner. Using PARP-1 mutants the importance of the first Zn-finger structure in the annealing process was confirmed (Table 2). There are contradictory opinions about the biological role of the two Zn-fingers present in PARP-1. Recently Langelier *et al.* have shown [48], [49] that the first Zn-finger participates in DNA binding and is solely responsible for the DNA-induced enzyme activity increase of PARP-1. The second Zn-finger displays a nearly hundred fold lower DNA binding constant but is avoidable for the increase of enzyme activity. In *Drosophila melanogaster* a new mutant form of PARP-1 has been found which lacks the first Zn-finger structure [50]. Through this mutant the transactivational and transrepressional functions of PARP-1 are uncoupled [50].

When the melting curve of the h *c-myc* FRET oligonucleotide was recorded *in vitro*, large oscillatory movements were detected between 25 and 35 Celsius degrees in the absence of h PARP-1. These movements were silenced in the presence of h PARP-1 (Fig. 4). Because changes in FRET can be connected to the increasing or decreasing distances of the two fluorophores we conclude that PARP-1 participates in a dynamic interconversion of isomorphic structures through the defolding process. This result also underlines the role of h PARP-1 in the regulation of *c-myc* expression.

The participation of PARP-1 in the regulation of transcription is firmly established and shown to happen at different steps of transcription. Chip-on-chip experiments have shown that PARP-1 DNA binding is enriched in 90% of the promoter regions of the actively transcribed genes by RNA polymerase II [51]. Maybe this value is a little bit overestimated, because others have shown more limited contribution of PARP-1 to the regulation of transcription.[52], [53] It has also been described, that the PARP-1 – Topoisomerase complexes participate in the initiation of transcription of a certain segment of genes [54]–[56]. It has been previously shown that PARP-1 can regulate the enzymatic activity of Topoisomerase I [57], [58]. When the h *c-myc* GQ structure binding proteins were isolated, the presence of Topoisomerase I was shown by immunoblotting in the eluate (Fig. S1B) of the affinity pull-down experiments. We have also observed during the FRET experiments that the added human Topoisomerase I protein increased the rate of DNA strand annealing, just as h PARP-1 did. When added together the rate increasing effect was additive and not synergistic (Table 2). We surmise that the two proteins acted independently of each other, and the annealing rate enhancing effect was not a consequence of their complex formation.

The best known model for the regulation of h *c-myc* gene's transcription through the GQ structures present in the NHE III_1_ part of the promoter has been crystallized out by the research group led by LH Hurley [3]–[8], [27], [33], [46]. Analyzing only the role of participating proteins it has been shown in both *in vivo* and *in vitro* experiments that nucleolin stabilizes the erected GQ structure [11], [27]. Contrary to that human NM23 H2, a nucleotide diphosphate kinase, binds to both of the single strands and unfolds those in a stepwise way, fulfilling a role PARP-1 might has [12]. The difference between NM23 H2 and PARP-1 is that PARP-1 is able to bind to the GQ structure and converting it into double-stranded B-DNA form *in vitro* in the presence of 100 mM KCl which concentration prevents the unfolding activity of NM23 H2. Other difference is that PARP-1 binds to the B-DNS form of the GQ structure too. Whether NM23 H2 and PARP-1 cooperate in the unfolding of the *c-myc* GQ structure is not known. But it has been shown that both proteins are members of the B-cell specific SWAP complex, participating in switching B-cell lines [59]. Moreover Khang *et al.* described a PAR-dependent E3 ubiquitin ligase complex, regulating DNA damage, named Iduna [60]. Iduna interacts with proteins which are either PAR binders or are PAR-ylated. Nucleolin was shown one of these proteins [60]. These facts underline the possibility of interaction of proteins in the regulation of *c-myc* expression. HnRNP K1 and CNBP are also proteins participating in the regulation of *c-myc* transcription [10], [14].

The experimental results described in this paper strongly supports, that h PARP-1 participates in the regulation of the expression of the h *c-myc* gene. Because c-MYC influences the expression of 10–15% of the genes present in the genome, this interaction partly might shed light onto the hitherto unexplained pleiotropic cell physiological behavior of the PARP-1 protein.

## Materials and Methods

HL60 (established human promyelocitic leukemia cell line), HeLa (established human cervical cancer cell line) and PARP −/− MEF (immortalized mouse embryonic fibroblast cell line deriving from PARP−/− mouse embryos) cells were grown in RPMI 1640 medium supplemented with 10% FBS and 100 Unit/ml of penicillin and 100 µg/ml streptomycin. While HL60 and HeLa cells were from ATCC, PARP−/− MEF cells is a genereous gift of Dr Erwin F. Wagner (IMP, Vienna, Austria). Oligonucleotides were obtained either from ITD or from Genosys. GQ structure forming oligonucleotides were incubated at 353 K for one minute in the presence of 100 mM of K^+^ to form the GQ structure.

Human recombinant PARP-1 and its truncated mutants were cloned into the baculovirus transfer vector pVL1392 and expressed in the baculovirus expression system of Pharmingen [61]. To produce recombinant viruses Sf9 cells were transfected with a mixture of Baculogold DNA (0.5 µg, Pharmingen) and 10 µg PARP-1 DNA cloned into the pVL1392 vector with help of Superfect dendritic liposome system (Quiagen) as suggested by the vendor. Recombinant viruses were produced by homologous recombination, the titer of the viruses were amplified until it reached a 10^8^ pfu/ml value and mass produced. Then Sf9 cells were infected with low MOI number recombinant viruses and cells were cultured for three days. Collected cells were disrupted by sonic disintegration, centrifuged at 20000×*g* for 15 minutes and supernatants were chromatographed on a 3-amino-benzamide-Sepharose column. PARP-1 containig fractions were loaded onto a hydroxyapatite column and separated from DNA contaminations as described earlier. Finally PARP-1 or mutant PARP-1 containing fractions were concentrated in Centricon YM50 cartridges. Besides the wild type PARP-1, the following truncated mutants were obtained: PARP-1 ΔZn-I, which starts at Gly93 (wild type numbering) and lacks the first Zn-finger domain; PARP-1 ΔZn-II, has the first Zn-finger, but the peptide sequence from His53 to His159 is omitted, therefore it lacks the second Zn-finger and the short peptide sequence connecting the two Zn-fingers; and PARP-1 ΔZn-I&II which starts at Lys253 and lacks both the Zn-fingers and the first nuclear localization signal too. Wild type PARP-1, and mutants PARP-1 ΔZn-I and PARP-1 ΔZn-I&II were obtained by PCR technology using Pfu DNA polymerase and wild type PARP-1 cDNA subcloned into vector pET21b. The sequences of the forward primers are, wild type: 5′ AGC T*GC GGC CGC* **ATG** GCG GAG TCT TCG GA; PARP-1 ΔZn-I: 5′ G ACA *GCG GCC GC*T **ATG** GGA GTG ACA GGC; PARP ΔZn-I&II: 5′ CAT C*GC GGC CGC* **ATG** AAG AAA GTG TGT. The reverse primer sequence is: 5′ C CTC *CCC GGG* TTA CCA CAG GGA GGT CTT. The recognition sites for NotI (forward) and for SmaI (reverse) are shown in italics and the positions of the start codons are underlined. To obtain the PARP-1 ΔZn-II mutant the pET21b clone was digested with KpnI which has two cutting sequences in the PARP-cDNA [62], vector and digest were separated in agarose gel, purified and truncated vector was religated. The removed fragment covers the second Zn-finger domain (His53 to His159). The truncated cDNA of PARP-1 was subcloned into the pVL1392 vector using the wild type primers in the PCR reaction. Vector constructs were DNA sequenced to confirm mutations.

### PARP activity measurements [26], [61], [63]

One pmol of h PARP-1 was incubated in a final volume of 20 µl with 20 µM activating nucleic acid and with 75 µM of ^3^H-NAD (60 dpm/pmol) in a solution containing 100 mM TRIS-HCl pH 7.2 buffer, 4 mM spermine for ten minutes. To quench the reaction 10% TCA was added and the precipitated proteins were filtered onto Whatmann GF/C filters, washed with TCA and finally with ethanol and dried. Radioactivity incorporated was assayed by liquid scintillation spectrometry. Results are expressed in pmol ADP-ribose incorporated/pmol of PARP-1× min units.

As an alternative method, one pmol of h PARP-1 was incubated under the conditions described above, but with 75 µM biotinylated-NAD as substrate and with different concentrations of h *c-myc* GQ DNA present. Incubation was ended by admixing identical volume of Laemmli sample buffer. Samples were loaded onto 10% SDS-PAGE gel, electrophoresed and transblotted onto nitrocellulose membrane. After blocking the membrane with 3% defatted milk dissolved in PBS, the blot was incubated with streptavidine-HPO conjugate (1 µg/ml) for an hour, followed by ECL detection of the incorporated biotin-ADP-ribose. Incorporation was quantitated using a BioRad image analyzer and results are given in pixel intensity/pmol PARP-1× min units.

In the binding assays, aliquots containing 0.45 pmols of human recombinant PARP-1 were incubated with 5 pmols of different 5′-biotin labeled DNA oligonucleotides in a final volume of 10 µl in a solution containing 20 mM TRIS-HCl, pH 7.4, 100 mM KCl and 2 mM of DTT (buffer A). At the end of incubation 1 µl of 0.5% formaldehyde was added and further incubated for 10 minutes then quenched by adding 1 µl of 1 M glycine solution. Two µl aliquots were spotted onto a nitrocellulose sheet, blocked and washed in 3% milk dissolved in PBS. PARP-1 bound oligonucleotides were detected by incubating the blots with HPO tagged streptavidine (1 µg/ml) and visualized by ECL. For quantitation, autoradiograms were analyzed by image analysis and results are given as % of control. During the binding experiments the following h *c-myc* GQ oligonucleotides were used: wild type GQ, 5′-biotin-TGG GGA GGG TGG GGA GGG TGG GGA AGG mutant-1 GQ, 5′-biotin-TGG GGA GGG TG**A** GGA GGG TGG GGA AGG mutant-2 GQ, 5′-biotin-TG**A** GGA GGG TGG GGA G**A**G TGG GGA AGG h-telomer, 5′-biotin-TTA GGG TTA GGG TTA GGG TTA GGG Bolded letters indicate the sites of mutations.

To carry out the pull-down assays, HeLa cells (20 millions) were starved in 1% FBS containing medium for 16 hours. To half of the cells 10% FBS was added and incubated further for 1.5 hours. In both cases ten millions of cells were harvested, washed with PBS twice and were disrupted by sonication. Then NP-40 in a final concentration of 1% was admixed and further incubated for 15 minutes on ice. After centrifugation (12000*× g* for 15 minutes at 4°C) aliquots equivalent to two million cells were incubated with 5 µl of the human wild type biotin-*myc* GQ DNA (250 µM) or with the same amount of human wild type biotin-*myc* GQ DNA converted previously into B-DNA form, or with biotin-tagged ds-dT/dA 25 mers on ice for an hour. Then 20 µl portions of streptavidine-agarose were admixed and incubated further for 30 minutes with shaking on ice. The resins were exhaustively washed with buffer A (10 ml aliquots of each washing steps) three times and finally twice with PBS. To the resins 100 µl of Laemmli sample buffer were added, boiled and 20 µl aliquots were loaded onto a 10% SDS-PAGE gel, electrophoresed and stained with colloidal Coomassie Brillant Blue [64]. Selected protein bands were cut out, the proteins were reduced, alkylated and in-gel digested with trypsin as described earlier [65]. The tryptic digests were analyzed by LC-MS/MS using an LCQ Fleet 3D ion trap mass spectrometer (Thermo Fischer Scientific) in a data-dependent fashion, in triple-play mode. Data analysis was performed by our Mascot in-house database search engine (ver.:2.2.04), against the NCBInr 100220 (183553 sequences) database, containing only human sequences. Proteins represented by at least 2 unique sequences with a score>40, i.e. with p<0.05, are reported [65]. The same, eluted protein fractions were also analyzed by immuno-blotting, using the following specific antibodies: h PARP-1 (Santa Cruz; sc-7150, H250), h Topoisomerase I (Topogen; 2012-1). Immunoblots were visualized by ECL [63].

FRET experiments were carried out as described earlier [57], using the human wild type *c-myc* GQ sequence containing DNA 27 mers [46], (5′-F- TGG GGA GGG TGG GGA GGG TGG GGA AGG-R), tagged at their 5′ end with fluoresceine and at their 3′ end with TAMRA. FRET experiments were carried out in a final volume of 50 µl containing 25 pmols of oligonucleotide in buffer A containing 1 mM of MgCl_2_. While excitation was at 488 nm, the emission spectra were recorded between 500–650 nms. In certain experiments the temperature of the holding cuvette was continuously increased with a rate of 2°C/2 minutes and emission readings were taken at 585 nm while exciting the system at 488 nm.

Luciferase reporter assays were done as follows: logarithmically growing PARP −/− MEF cells were transfected with 25 µg of Del-4-*myc-luciferase* reporter plasmid (a generous gift of Dr. Bert Vogelstein) [66] and with 5 µg of pcDNA3.1.-*beta-galactosidase* plasmid (generous gift of Dr. Maria Sasvari) in 0.5 ml of complete medium together with Superfect reagent (25 µl), for six hours together with or without 10 µg of pcDNA3.1-h-*parp-1* plasmid. After 28 hours cells were trypsinized and subdivided into 24 well plates. Seventy-two hours after transfection cells were lysed (Sigma Luciferase Kit) and the luciferase and the beta-galactosidase activities of samples were determined as suggested by the vendor. The luciferase activities were normalized to the *beta-galactosidase* expressions. Assays were carried out in six parallels. The expression of PARP-1 was detected by Western blotting.

Chromatin immunoprecipitation (ChIP) was carried out as recommended by the vendor of the kit (Upstate) and using five millions of logarithmically growing or DMSO treated (1 or 24 hours of treatment) HL60 cells or HeLa cells grown in the presence of 10% FBS or starved in 1% FBS for 16 hours [67]. In another set of experiments cells (five millions/immunoprecipitation) were first *in vivo* crosslinked with 2 mM of EGS (Pierce) for 25 minutes, before the addition of 1% of formaldehyde [37]. Immunoprecipitation and isolation of DNA was described as above. In the ChIP experiments two antibodies raised against PARP-1 protein were used, providing the same results. The H250 is a polyclonal rabbit antibody against PARP-1, and was applied in a dilution of 2 µg antibody per extract of one million cells. The mouse monclonal antibody C 2–10 was used in a concentration of 2 µl ascites fluid per extract of one million cells. Both antibodies are the product of Santa Cruz.

Thorough the experiments statistical analysis was carried out using paired Students t-test. * means significantly different, p<0.05

## Supporting Information

Figure S1 **Protein profile of h HeLa cell proteins obtained in** ***in vitro*** **affinity pull-down experiments carried out under various conditions.** Panel A The protein profile of DNA bound proteins obtained from HeLa cells. HeLa cells were cultured either in 10% FBS (lanes 4, 5, 6, 7) or in 1% FBS (lanes 8, 9, 10, 11) containing RPMI 1640 medium. Biotin-h-c-*myc* GQ (lanes 4, 8), biotin-double stranded h-*c-myc* GQ (lanes 5, 9); biotin-dAdT (lanes 6, 10) and no added DNA (lanes 7, 11) were applied as baits and oligonucleotide-bound proteins were pulled down with streptavidine-agarose. Proteins were visualized by colloid Coomassie Blue staining. Lane 1 shows the protein profile of HeLa cells grown in 10% FBS containing medium, lane 2 the same for 1% FBS starved HeLa cells and lane 3 shows the protein profile of the control experiment, where no cell extract was added. MW shows the distribution of molecular weight markers. Arrows indicate the protein bands cut out and sent for MS sequencing. Panel B Western blot of proteins present in lane 4 and 5 in Supplementary Fig. 1A and probed with different antibodies. Immunoblots were carried out with antibodies developed against PARP-1, Topoisomerase I (lane 1, control where an aliquote from the last washing fraction from the pull-down experiment was loaded onto the SDS-PAGE gel; lane 2, fraction 4; lane 3, fraction 5).
(TIF)

Figure S2 **RT-qPCR analysis of the** ***in vivo*** **expression of the** ***c-myc*** **,** ***cyclin D*** **,** ***parp-1*** **and** ***gapdh*** **genes in starved and logarithmically growing HeLa cells.** HeLa cells were kept starving in 1% FBS containing medium for 16 hours, then half of the cells were refed with 10% FBS containing medium and cultured for one hours. Total RNAs were isolated (Qiagen RNeasy kit) from ten million cells from each cell populations and equal amounts of RNAs were reverse transcribed into cDNA (BioRad iScript kit). Equal volumes from both samples were analyzed for gene expressions using qPCR. PCR products were electrophoresed in 1.7% agarose gels, EtBr-stained and visualized in UV light and are shown in the figure. On the left side of the picture molecular weight markers are shown. The following PCR primers were used *cmyc*F: 5′ GGT CTT CCC CTA CCC TCT CAA, *cmyc*R: 5′CGT TTG TGT GTT CGC CTC TTG; *parp-1*F: 51 GTG TGG GTA CGG TGA TCG GTA, *parp-1*R: 5′ GCC TGC ACA CTG TCT GCA TT; *cycD*F: 5′ CCC GCT GGC CAT GAA CTA, *cycD*R: 5′ CGG AGG CAG TCT GGG TCA; and *gapdh*F: 5′ GAA GGT GAA GGT CGG AGT C, *gapdh*R: 5′ GAA GAT GGT GAT GGG ATT TC.
(TIF)

Figure S3 **ChIP-qPCR experiment shows the** ***in vivo*** **binding of h PARP-1 to the promoter region of h** ***c-myc*** **gene.** Chromatin immunoprecipitation experiments were carried out, applying a PARP-1 antibody (Santa Cruz, H250, 2 µg/extract of one million cells), both in growing HL60 cells or in HL60 cells treated for two hours with 1.7% of DMSO to induce differentiation. Isolated DNAs were the subject of qPCR analysis with primers specific to the promoter region of the *c-myc* gene. A typical pair of PCR curves is shown, where on the ordinate the measured EvaGreen fluorescence values are shown after each PCR cycle and where the abscissa shows the number of PCR cycles. The sequences of the primers are: MQ1F: 5′ GAC AAG GAT GCG GTT TGT CA; MQ1R: 5′ CTC TCG CTG GAA TTA CTA CAG CG [39].
(TIF)

Figure S4 **The FRET activity of F-** ***c-myc*** **GQ-R molecule, determined in the absence and in the presence of h PARP-1. Effect of the complementary strand oligonucleotide.** 1 µg of h PARP-1 was incubated with the F-h-*c-myc* GQ-R oligonucleotide present in the GQ structure form in the absence or in the presence of the complementary oligonucleotide strand (10 fold molar excess) for three minutes, than the FRET intensities were recorded between 500 and 650 nm, while excitation was at 485 nm.
(TIF)

Table S1 **Effect of various polynucleotides on the enzymatic activity of h PARP-1.** One picomole of PARP-1 was incubated with 75 µM of [^3^H]-NAD (specific activity was 60 dpm/pmol) in the presence of various oligonucleotides (20 µM) for 10 minutes. After incubation 10% TCA was admixed and the precipitated proteins were filtered on Whatman-GFC filters. Incorporated radioactivity was determined by liquid scintillation spectrometry. Average values of triplicates are shown, where standard deviation is less than 10%. Results are expressed as pmol ADP-ribose incorporated/pmol PARP-1× min values.
(PDF)

Table S2 **Isolation and analysis of HeLa cell proteins with binding affinity towards the h** ***c-myc*** **GQ structure under** ***in vitro*** **conditions.** Affinity pull-down experiments were carried out using single- and double-stranded forms of biotin-h-*c-myc* GQ and ds-biotin-dAdT DNA as baits to bind proteins present in HeLa cell extracts. The isolated proteins were separated by SDS-PAGE. Selected protein bands were cut out from colloidal Coomassie Blue-stained gels, trypsin digested and MS sequenced to identify the proteins. Table S2 lists the isolated, sequenced, co-migrating proteins as indicated by the arrows in Fig. S1.
(PDF)
