# Supplementary figures and images for: The Guanine-Quadruplex Structure in the Human c-myc Gene's Promoter Is Converted into B-DNA Form by the Human Poly(ADP-Ribose)Polymerase-1
Source: PLoS One. 2012 Aug 6;7(8):e42690. doi: 10.1371/journal.pone.0042690 (PMC3412819; doi:10.1371/journal.pone.0042690)

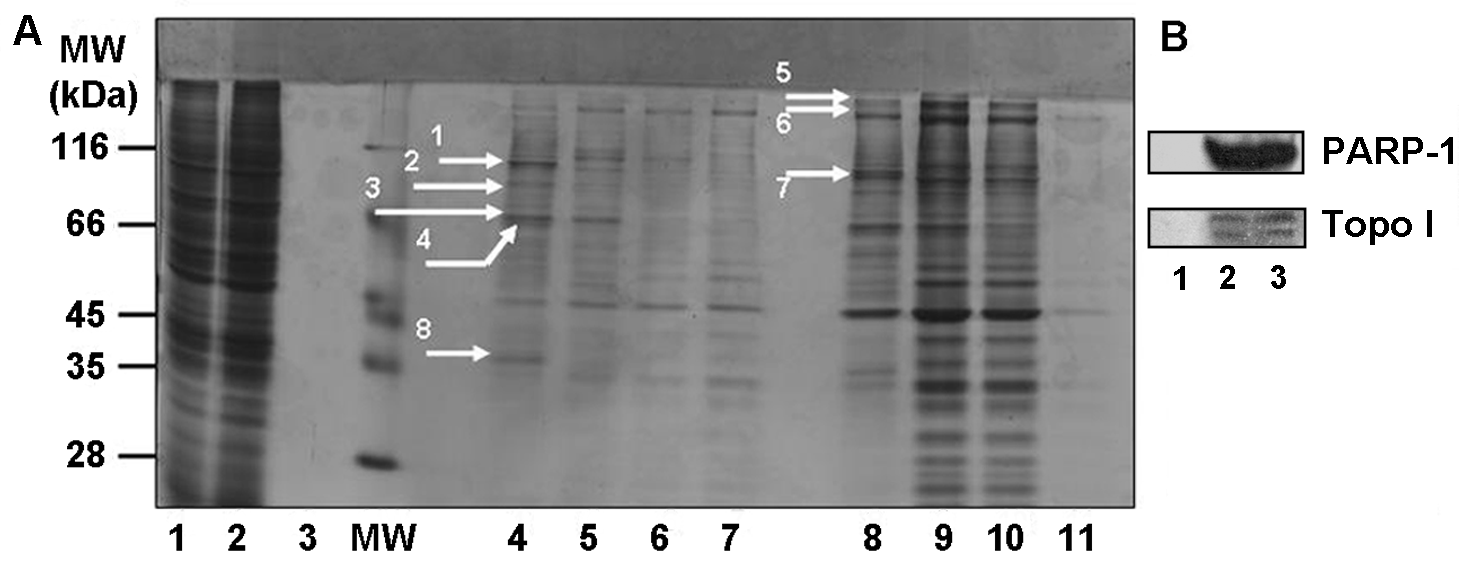

Supplement: Figure S1 — Protein profile of h HeLa cell proteins obtained in in vitro affinity pull-down experiments carried out under various conditions. Panel A The protein profile of DNA bound proteins obtained from HeLa cells. HeLa cells were cultured either in 10% FBS (lanes 4, 5, 6, 7) or in 1% FBS (lanes 8, 9, 10, 11) containing RPMI 1640 medium. Biotin-h-c-myc GQ (lanes 4, 8), biotin-double stranded h-c-myc GQ (lanes 5, 9); biotin-dAdT (lanes 6, 10) and no added DNA (lanes 7, 11) were applied as baits and oligonucleotide-bound proteins were pulled down with streptavidine-agarose. Proteins were visualized by colloid Coomassie Blue staining. Lane 1 shows the protein profile of HeLa cells grown in 10% FBS containing medium, lane 2 the same for 1% FBS starved HeLa cells and lane 3 shows the protein profile of the control experiment, where no cell extract was added. MW shows the distribution of molecular weight markers. Arrows indicate the protein bands cut out and sent for MS sequencing. Panel B Western blot of proteins present in lane 4 and 5 in Supplementary Fig. 1A and probed with different antibodies. Immunoblots were carried out with antibodies developed against PARP-1, Topoisomerase I (lane 1, control where an aliquote from the last washing fraction from the pull-down experiment was loaded onto the SDS-PAGE gel; lane 2, fraction 4; lane 3, fraction 5). (TIF) [file pone.0042690.s001.tif]

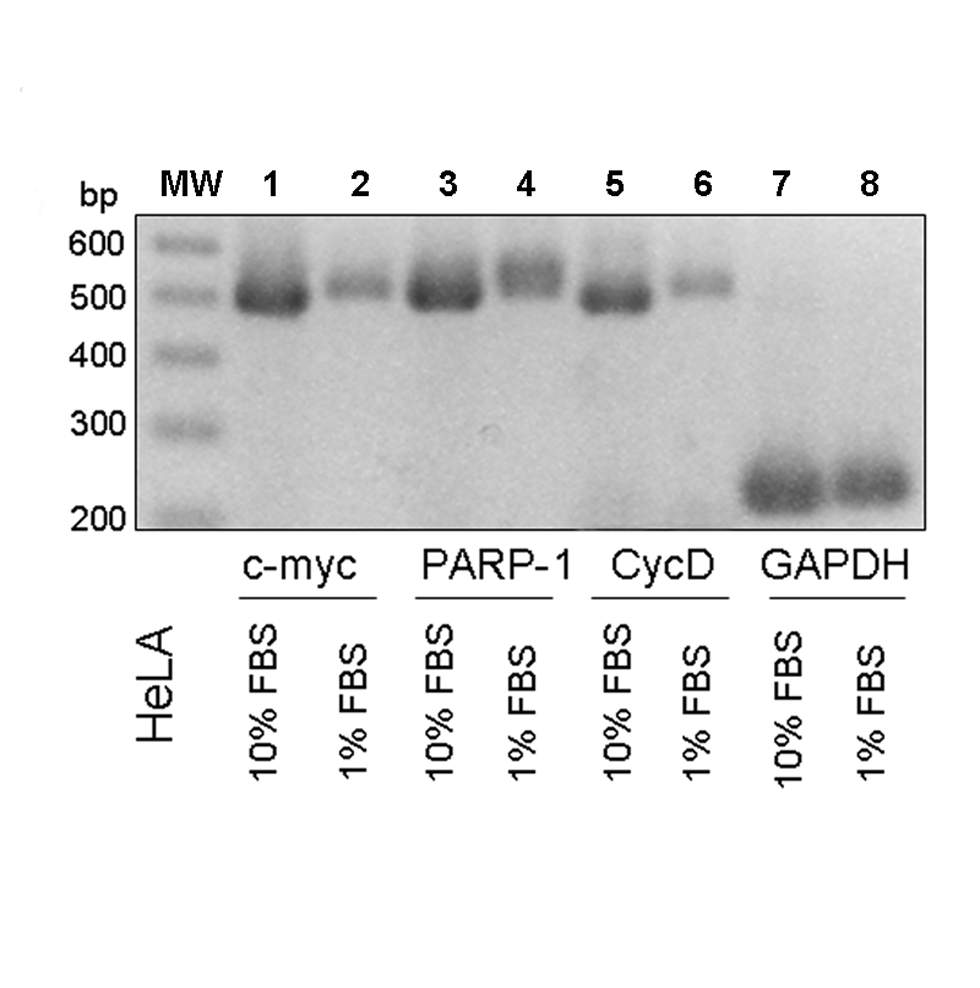

Supplement: Figure S2 — RT-qPCR analysis of the in vivo expression of the c-myc , cyclin D , parp-1 and gapdh genes in starved and logarithmically growing HeLa cells. HeLa cells were kept starving in 1% FBS containing medium for 16 hours, then half of the cells were refed with 10% FBS containing medium and cultured for one hours. Total RNAs were isolated (Qiagen RNeasy kit) from ten million cells from each cell populations and equal amounts of RNAs were reverse transcribed into cDNA (BioRad iScript kit). Equal volumes from both samples were analyzed for gene expressions using qPCR. PCR products were electrophoresed in 1.7% agarose gels, EtBr-stained and visualized in UV light and are shown in the figure. On the left side of the picture molecular weight markers are shown. The following PCR primers were used cmycF: 5′ GGT CTT CCC CTA CCC TCT CAA, cmycR: 5′CGT TTG TGT GTT CGC CTC TTG; parp-1F: 51 GTG TGG GTA CGG TGA TCG GTA, parp-1R: 5′ GCC TGC ACA CTG TCT GCA TT; cycDF: 5′ CCC GCT GGC CAT GAA CTA, cycDR: 5′ CGG AGG CAG TCT GGG TCA; and gapdhF: 5′ GAA GGT GAA GGT CGG AGT C, gapdhR: 5′ GAA GAT GGT GAT GGG ATT TC. (TIF) [file pone.0042690.s002.tif]

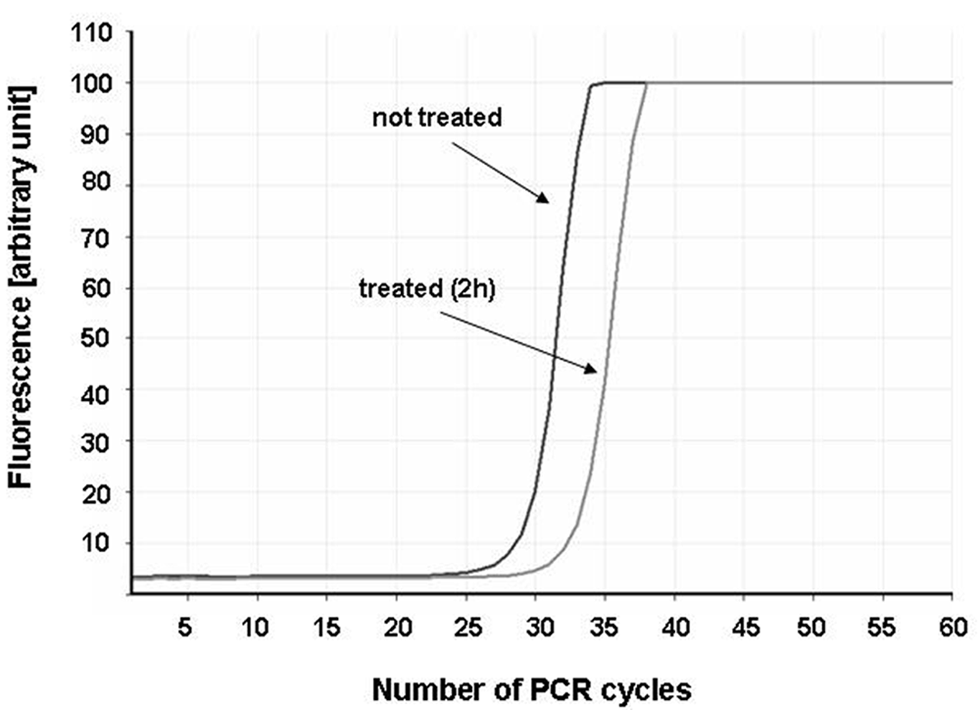

Supplement: Figure S3 — ChIP-qPCR experiment shows the in vivo binding of h PARP-1 to the promoter region of h c-myc gene. Chromatin immunoprecipitation experiments were carried out, applying a PARP-1 antibody (Santa Cruz, H250, 2 µg/extract of one million cells), both in growing HL60 cells or in HL60 cells treated for two hours with 1.7% of DMSO to induce differentiation. Isolated DNAs were the subject of qPCR analysis with primers specific to the promoter region of the c-myc gene. A typical pair of PCR curves is shown, where on the ordinate the measured EvaGreen fluorescence values are shown after each PCR cycle and where the abscissa shows the number of PCR cycles. The sequences of the primers are: MQ1F: 5′ GAC AAG GAT GCG GTT TGT CA; MQ1R: 5′ CTC TCG CTG GAA TTA CTA CAG CG [39]. (TIF) [file pone.0042690.s003.tif]

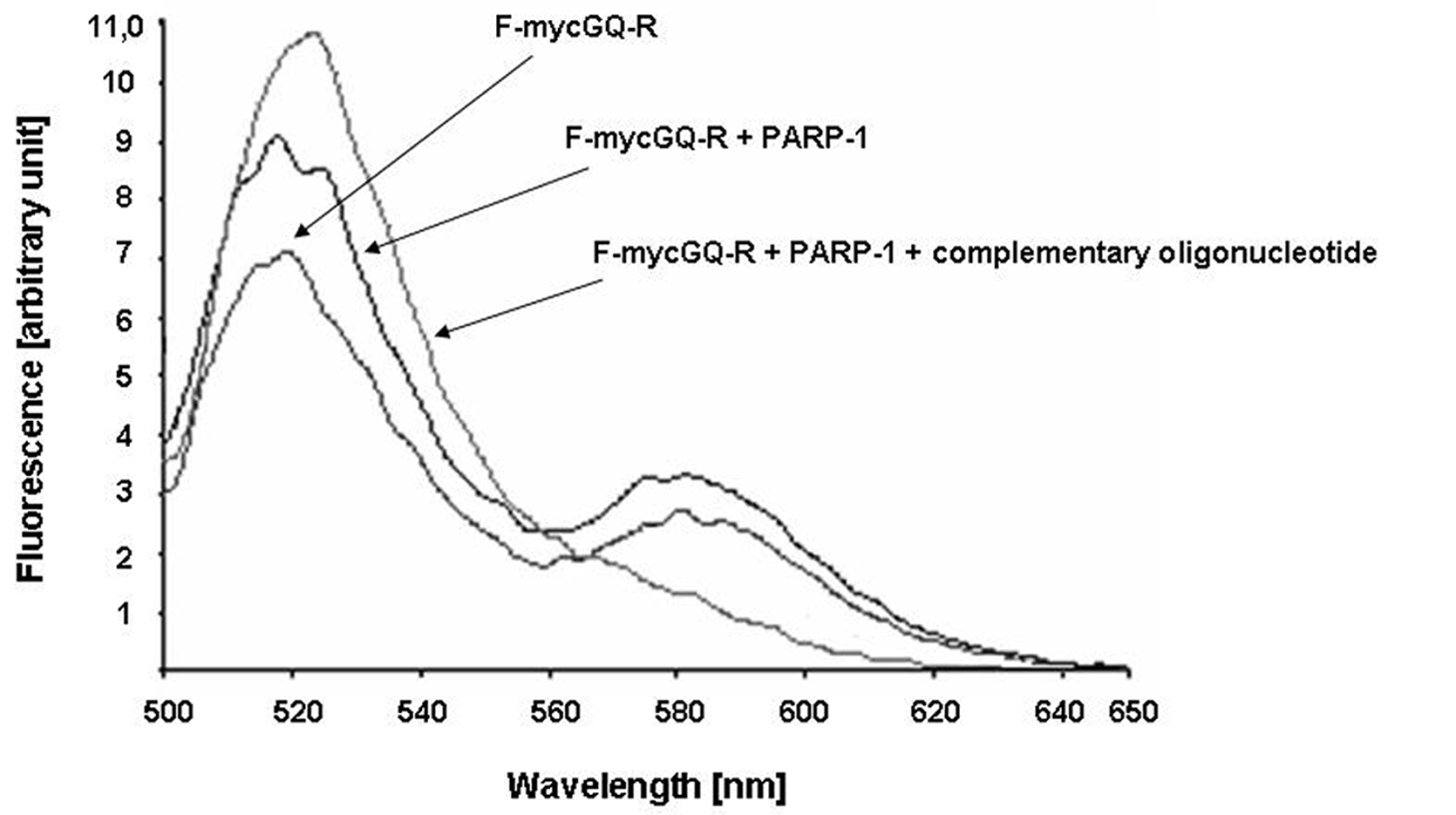

Supplement: Figure S4 — The FRET activity of F- c-myc GQ-R molecule, determined in the absence and in the presence of h PARP-1. Effect of the complementary strand oligonucleotide. 1 µg of h PARP-1 was incubated with the F-h-c-myc GQ-R oligonucleotide present in the GQ structure form in the absence or in the presence of the complementary oligonucleotide strand (10 fold molar excess) for three minutes, than the FRET intensities were recorded between 500 and 650 nm, while excitation was at 485 nm. (TIF) [file pone.0042690.s004.tif]
