# Supplementary material for: The Guanine-Quadruplex Structure in the Human c-myc Gene's Promoter Is Converted into B-DNA Form by the Human Poly(ADP-Ribose)Polymerase-1
Source: PLoS One. 2012 Aug 6;7(8):e42690. doi: 10.1371/journal.pone.0042690 (PMC3412819; doi:10.1371/journal.pone.0042690)
Supplement: Table S1 — Effect of various polynucleotides on the enzymatic activity of h PARP-1. One picomole of PARP-1 was incubated with 75 µM of [3H]-NAD (specific activity was 60 dpm/pmol) in the presence of various oligonucleotides (20 µM) for 10 minutes. After incubation 10% TCA was admixed and the precipitated proteins were filtered on Whatman-GFC filters. Incorporated radioactivity was determined by liquid scintillation spectrometry. Average values of triplicates are shown, where standard deviation is less than 10%. Results are expressed as pmol ADP-ribose incorporated/pmol PARP-1× min values. (PDF) [file pone.0042690.s005.pdf]

Supplementary Table 1 Effect of various polynucleotides on the enzymatic activity of PARP-1.

Modulator added	Incorporation (pmolADP-ribose/pmol PARP-1 x min)	Incorporation (%)
dAdT	58.7	100
gap DNA	37.9	65
loop DNA	35.6	61
myc-GQ DNA	12.0	20
dA or dT	not detected	0
loop RNA	not detected	0
ds RNA (23 bp)	not detected	0
polyrA	0.95	1,6
polyrI	1.4	2,3
polyrG	1.1	1,8
