# Supplementary material for: The Guanine-Quadruplex Structure in the Human c-myc Gene's Promoter Is Converted into B-DNA Form by the Human Poly(ADP-Ribose)Polymerase-1
Source: PLoS One. 2012 Aug 6;7(8):e42690. doi: 10.1371/journal.pone.0042690 (PMC3412819; doi:10.1371/journal.pone.0042690)
Supplement: Table S2 — Isolation and analysis of HeLa cell proteins with binding affinity towards the h c-myc GQ structure under in vitro conditions. Affinity pull-down experiments were carried out using single- and double-stranded forms of biotin-h-c-myc GQ and ds-biotin-dAdT DNA as baits to bind proteins present in HeLa cell extracts. The isolated proteins were separated by SDS-PAGE. Selected protein bands were cut out from colloidal Coomassie Blue-stained gels, trypsin digested and MS sequenced to identify the proteins. Table S2 lists the isolated, sequenced, co-migrating proteins as indicated by the arrows in Fig. S1. (PDF) [file pone.0042690.s006.pdf]

Supplementary Table 2 Isolation and analysis of c-myc-GQ binding proteins from HeLa cells.

Protein band (Fig S1A)	NCBI Acc. #	MW*/ Da	C%†	Unique peptides	Protein name
1	gi|189306	76355	22	13	nucleolin
	gi|190267	113805	2	2	poly(ADP-ribose) polymerase
2	gi|306891	83584	12	8	90kDa heat shock protein
	gi|61656603	98622	7	5	heat shock protein HSP 90-alpha 2
	gi|5031755	71184	5	3	heterogeneous nuclear ribonucleoprotein R isoform 2
3	gi|5729877	71082	9	7	heat shock 70kDa protein 8 isoform 1
	gi|386785	70110	9	5	heat shock protein
	gi|62089046	65496	19	10	heterogeneous nuclear ribonucleoprotein M isoform a variant
	gi|4503841	70084	10	5	ATP-dependent DNA helicase II, 70 kDa subunit
4	gi|5031703	52189	20	6	Ras-GTPase-activating protein SH3-domain-binding protein
	gi|226021	67394	12	5	growth regulated nuclear 68 protein
5	gi|179106	284905	10	22	nonerythroid alpha-spectrin
	gi|338443	275259	12	19	beta-spectrin
	gi|53791219	280069	3	6	filamin A
6	gi|12667788	227646	19	34	myosin, heavy polypeptide 9, non-muscle
	gi|641958	229824	3	5	non-muscle myosin B
7	gi|190267	113805	23	18	poly(ADP-ribose) polymerase
	gi|189306	76355	15	9	nucleolin
	gi|167830433	115600	4	3	DEAH (Asp-Glu-Ala-His) box polypeptide 36 isoform 1
8	gi|4504445	34289	20	6	heterogeneous nuclear ribonucleoprotein A1 isoform a
	gi|4557032	36900	21	7	L-lactate dehydrogenase B

* calculated from the database sequence; this number may be different from the size of the active form of the protein

† sequence coverage based on the above sequence
